# Botulinum Toxin Injections for Psychiatric Disorders: A Systematic Review of the Clinical Trial Landscape

**DOI:** 10.3390/toxins16040191

**Published:** 2024-04-15

**Authors:** Ilya Demchenko, Alyssa Swiderski, Helen Liu, Hyejung Jung, Wendy Lou, Venkat Bhat

**Affiliations:** 1 Interventional Psychiatry Program, St. Michael’s Hospital—Unity Health Toronto, 193 Yonge Street, Toronto, ON M5B 1M4, Canada; ilya.demchenko@unityhealth.to (I.D.); 19ams23@queensu.ca (A.S.); helen.liu2@mail.mcgill.ca (H.L.); 2 Institute of Medical Science, Temerty Faculty of Medicine, University of Toronto, 1 King’s College Circle, Toronto, ON M5S 1A8, Canada; 3 Institute for Biomedical Engineering, Science, and Technology (iBEST), Keenan Research Centre for Biomedical Science, St. Michael’s Hospital—Unity Health Toronto, 209 Victoria Street, Toronto, ON M5B 1T8, Canada; 4 Dalla Lana School of Public Health, University of Toronto, 155 College Street, Toronto, ON M5T 3M7, Canada; 5 Department of Psychiatry, Temerty Faculty of Medicine, University of Toronto, 250 College Street, Toronto, ON M5T 1R8, Canada; 6 Neuroscience Research Program, St. Michael’s Hospital—Unity Health Toronto, 209 Victoria Street, Toronto, ON M5T 3M7, Canada

**Keywords:** mental disorders, botulinum toxins, acetylcholine release inhibitors, facial expression, clinical trial, systematic review, Botox, neuromuscular agents, neurotoxins, cosmetic techniques

## Abstract

Botulinum toxin type A (BONT-A) has shown promise in improving the mood-related symptoms of psychiatric disorders by targeting muscles linked to the expression of negative emotions. We conducted a systematic review of past and ongoing efficacy trials of BONT-A therapy for psychiatric disorders to identify relevant trends in the field and discuss the refinement of therapeutic techniques. A comprehensive search for published clinical trials using BONT-A injections for psychiatric disorders was performed on 4 May 2023 through OVID databases (MEDLINE, Embase, APA PsycINFO). Unpublished clinical trials were searched through the ClinicalTrials.gov and International Clinical Trial Registry Platform public registries. The risk of bias was assessed using the JBI Critical Appraisal tools for use in systematic reviews. We identified 21 studies (17 published, 4 unpublished clinical trials) involving 471 patients. The studies focused on evaluating the efficacy of BONT-A for major depressive, borderline personality, social anxiety, and bipolar disorders. BONT-A was most commonly injected into the glabellar area, with an average dose ranging between 37.75 U and 44.5 U in published studies and between 32.7 U and 41.3 U in unpublished trials. The results indicated significant symptom reductions across all the studied psychiatric conditions, with mild adverse effects. Thus, BONT-A appears to be safe and well-tolerated for psychiatric disorders of negative affectivity. However, despite the clinical focus, there was a noted shortage of biomarker-related assessments. Future studies should focus on pursuing mechanistic explorations of BONT-A effects at the neurobiological level.

## 1. Introduction

Psychiatric disorders are prevalent and debilitating, and they often feature disturbances in mood and personality, along with a decreased interest in pleasurable activities [1]. Additionally, these disorders present physical signs, such as changes in body movements, speech, hygiene habits, weight, and motor activity, which often assist clinicians in making a correct diagnosis [2,3]. Frequently, psychiatric conditions are accompanied by specific facial expressions reflecting emotional experience [4]. The facial feedback hypothesis posits that one’s facial expression and the corresponding activity of the facial musculature modulate subjective experiences of emotion [5,6,7]. This hypothesis offers theoretical insights into the potential etiology and mechanisms of affective disorders. Given this perspective, disrupting this feedback loop at the neural level can be of therapeutic benefit.

One possible way of disrupting this limbic–motor arc is by inducing a temporary paralysis of the facial musculature through injections of the botulinum toxin type A (BONT-A) neurotoxic protein into the glabellar region of the face, which is responsible for the facial expression of frowning. BONT-A exerts its effects on presynaptic nerve terminals by blocking the release of acetylcholine into the synaptic cleft, thus preventing neurotransmission and, as a corollary, disrupting proprioceptive and interoceptive facial feedback mechanisms [8,9]. Several studies have examined the association between BONT-A injections and emotional processing, suggesting that BONT-A may be used as a potential treatment for affective disorders [10,11,12]. Putative neurobiological markers of treatment response to BONT-A have also been explored, with functional magnetic resonance imaging (fMRI) studies [13,14,15] providing evidence of successful attenuation of amygdala reactivity in response to angry faces upon paralysis of the facial muscles via BONT-A injection.

Since the initial case series of BONT-A therapy for major depressive disorder (MDD) published in 2006 [16], the efficacy and tolerability of a single BONT-A injection have been evaluated in male and female patients in various clinical trial contexts, including open-label and randomized controlled trials (RCTs). Most studies in the field, however, have explored its therapeutic benefits for MDD specifically. MDD-focused pooled [17] and meta-analyses [18,19,20,21] have consistently reported a 45–55% reduction in depressive symptoms, a 50–60% response rate, and approximately one-third of patients achieving remission after BONT-A injections, concluding that BONT-A has an overall positive effect on reducing depressive symptoms in comparison to placebo injections. These conclusions are supported by the large effect sizes reported in these meta-analyses (e.g., Hedge’s *g* = −0.82 [95% CI, −1.38 to −0.27] [20] or Cohen’s *d* = 0.98 [95% CI, 0.47 to 1.49] [18]). Currently, BONT-A injections into the facial muscles of the glabella represent a promising novel and well-tolerated treatment option for MDD that is being investigated in Phase III clinical trials. Furthermore, emerging studies seek to explore its efficacy in populations with other psychiatric disorders with a significant affectivity component and potentially overlapping neurobiological mechanisms.

As the application of BONT-A therapy in psychiatry continues to expand, the conduct of well-designed clinical trials with theory-informed research hypotheses becomes critical. Systematic reviews and meta-analyses, which aim to evaluate scientific evidence, typically rely on already published results [22]. In this way, the present-day understanding of the efficacy of BONT-A may be biased by overrepresented positive findings and limited knowledge of the treatment parameters, relying on those reported in studies that have been successfully published in peer-reviewed journals. Moreover, previous systematic reviews and meta-analyses on the topic have focused on the applications of BONT-A for MDD specifically [18,20], but given the transdiagnostic nature of network- and neuroimaging-based substrates of mood and emotion [23], novel studies examining the applications of BONT-A in the context of other affect-related psychopathologies are gaining traction. The current systematic review aims to comprehensively map the methodologies and findings from both published and registered but unpublished clinical trials that explore the use of BONT-A injections as a treatment option for various psychiatric disorders.

## 2. Results

**Figure 1:**
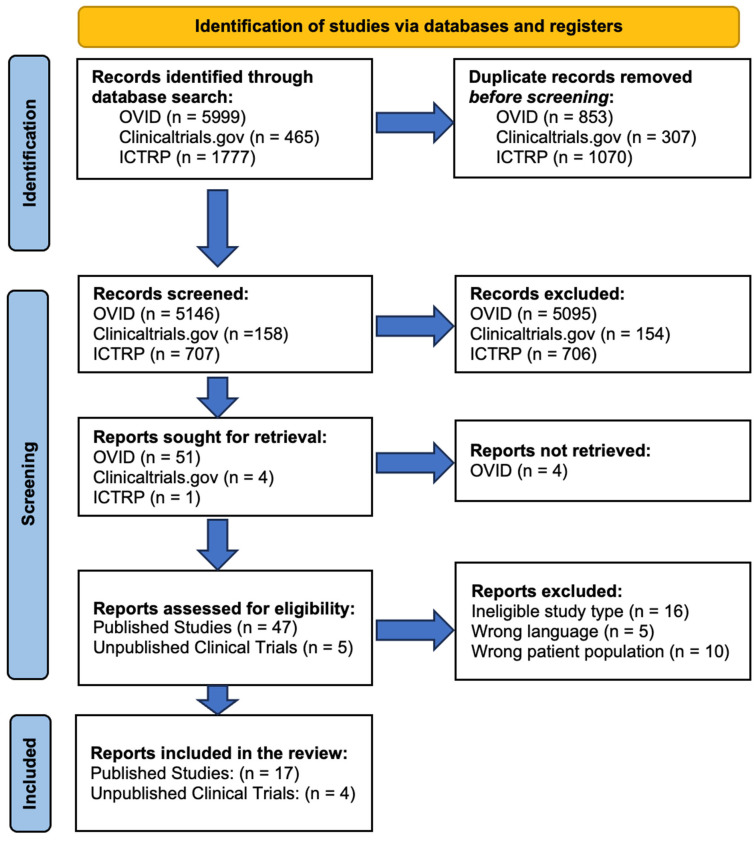
PRISMA flow chart reflecting the search and screening process of the published studies and unpublished registered clinical trials included in this systematic review of botulinum toxin type A therapy for psychiatric disorders.

The search of the three OVID databases (Embase, MEDLINE, APA PsycINFO) yielded 5146 results after removing duplicates (Figure 1). After screening for eligibility, 17 relevant published studies were included in this systematic review. The search on ClinicalTrials.gov and ICTRP yielded a combined total of 856 trials after duplicate removal. Following screening for eligibility, four unpublished clinical trials in total were included in this systematic review (Figure 1). Among the reviewed studies, one published study recruited both patients and healthy control groups.

### 2.1. Quality Assessment

Supplementary Tables S1–S3 present the outcomes of the quality assessment for the included studies. Eleven out of seventeen studies (64.7%) were randomized [15,24,25,26,27,28,29,30,31,32,33], whereas the other six non-randomized studies presented five case series (29.4%) [16,34,35,36,37] and one open-label trial (5.9%) [38]. Among the case series, the condition was measured in a standard and reliable way, the outcomes were clearly reported, and four out of five (80.0%) case series had clear and appropriate statistical analyses. However, only one out of five (20.0%) case series presented clear inclusion criteria for the participants or their clinical information, and none reported clear demographic information. For all the RCTs, the participants and those delivering the treatment were blinded in 8 out of 11 (72.7%) of the trials. Overall, the RCTs were well-designed and executed: all 11 RCTs (100.0%) utilized true randomization, included blinded outcome assessors, consistently and reliably measured the outcomes across the treatment groups, and used appropriate statistical analyses. Three RCTs (27.2%), however, were not clear about completing the follow-up. Overall, all 17 studies included in this systematic review measured outcomes consistently and reliably. All the studies were also deemed to have an appropriate study design. The only open-label trial [38] was overall well-conducted but was unclear with regard to the strategies for mitigating confounding variables and incomplete follow-up.

### 2.2. Studies by Start Date and Completion Status

**Figure 2:**
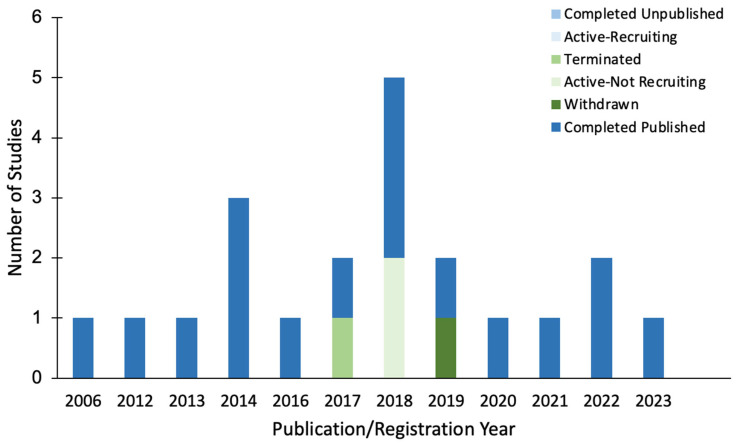
Bar graph displaying the publication or registration year of the included published studies and unpublished registered clinical trials, along with their corresponding completion status.

*Published Studies*. The first study investigating BONT-A injections for the treatment of psychiatric disorders was published in 2006, followed by the second study published in 2012 (Figure 2). Since 2012 and until 2023, studies examining the use of BONT-A for psychiatric disorders have been consistently published at an average rate of one per year.

*Registered Clinical Trials*. Of the four trials registered between 2017 and 2019, two trials (50.0%) were listed as active but not yet recruiting, one was terminated (25.0%), and one was withdrawn (25.0%) (Figure 2).

### 2.3. Studies by Phase and Design

**Figure 3:**
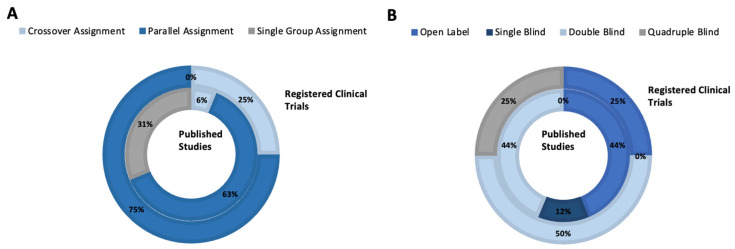
Sunburst charts illustrating the study design of included published studies and unpublished registered clinical trials. (**A**) Distribution of published studies and unpublished clinical trials by study allocation. (**B**) Utilization of masking in published studies and unpublished registered clinical trials.

*Published Studies*. All 17 published studies (100.0%) were interventional clinical trials. Eight of the studies (47.1%) were comparing active treatments to placebo, and within these, seven (41.2%) had two study arms. The remaining one study (5.9%) had four arms, wherein two different doses of BONT-A and a placebo treatment were compared. Five of the studies (29.4%) had no comparison, and the remaining study compared MDD patients with healthy controls. In terms of masking, seven of the studies (41.2%) were double-blind, seven studies (41.2%) were open-label trials, two were single-blind (11.8%), and the remaining one (5.9%) had unknown masking (Figure 3A). Ten studies (58.8%) used parallel assignment [13,14,15,17,18], five (29.4%) used single-group assignment, one study used crossover assignment (5.9%), and the remaining study (5.9%) had concealed assignment (Figure 3B).

*Registered Clinical Trials*. All four of the unpublished registered trials (100.0%) were categorized as interventional clinical trials. All of the included trials listed their allocation as randomized. One trial (25.0%) was open-label, two trials (50%) were double-blind, and one trial (25.0%) was quadruple-blind (Figure 3A). According to the records of the registries, three trials (75.0%) randomized the participants using parallel assignment, whereas one trial (25.0%) used crossover assignment (Figure 3B). Of the two trials that specified the phase, one trial (50.0%) was a Phase I trial, and the other (50.0%) was a Phase II–III trial. As part of the design, all four trials had two treatment arms: three (75.0%) of these trials performed an active vs. placebo comparison, and one trial (25.0%) had two arms comparing BONT-A injections into two distinct facial sites (i.e., glabellar area vs. crow’s feet area).

### 2.4. Studies by Clinical Indication

**Figure 4:**
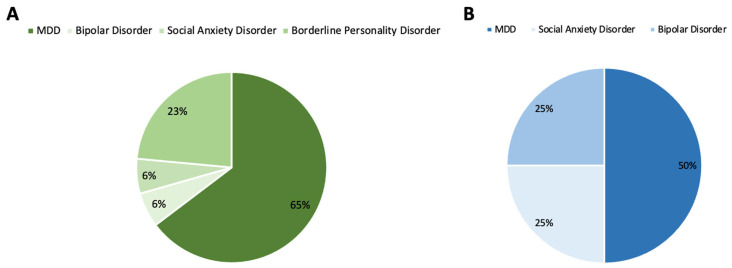
Pie charts depicting the primary clinical indications under investigation. (**A**) Primary indications of published studies. (**B**) Primary indications of unpublished registered clinical trials.

*Published Studies*. Most of the studies examined the applications of BONT-A for MDD (64.7%). Borderline personality disorder emerged as the second most researched indication (23.5%), followed by bipolar disorder (5.9%) and social anxiety disorder (5.9%) (Figure 4B).

*Registered Clinical Trials*. Most unpublished clinical trials also addressed MDD (50.0%), followed by bipolar disorder (25.0%) and social anxiety disorder (25.0%) (Figure 4B).

### 2.5. Studies by Enrolled Participants

**Figure 5:**
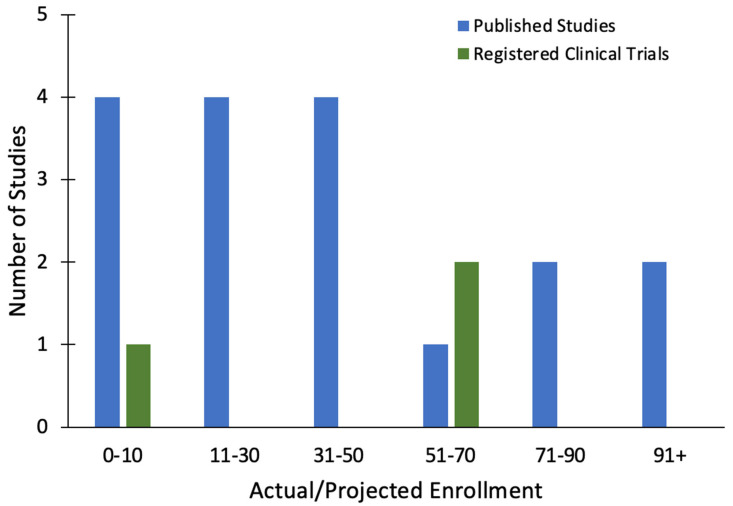
Bar graph illustrating the actual and projected enrollment numbers across the published studies and unpublished registered clinical trials.

*Published Studies*. There was significant variability in the sample sizes across the reviewed studies, ranging from 6 to 139 participants. Twelve (70.6%) of the published studies had a sample size between six and fifty participants (Figure 5). Irrespective of the assigned study arm, the median total enrollment size across all the studies was 42 participants, and the mean was 54 participants (standard deviation [*SD*] = 60). By the end of each study, the number of subjects who completed the treatment protocol was median = 42 and mean = 44 (*SD* = 34). Among them, the number of subjects allocated to the active BONT-A arm and who completed the treatment protocol was median = 24 subjects and mean = 29 subjects (*SD* = 22). Taken together, 918 participants were enrolled in all published studies to date, and 471 of the participants underwent BONT-A treatment.

Nine of the studies (52.9%) recruited both male and female participants, while the remaining eight (47.1%) recruited only female participants. The average inclusion age minimum and maximum were mean = 21.9 (*SD* = 6.3) and mean = 61.8 (*SD* = 13.3) years old, respectively. Across the 17 published studies, 15 of them (88.2%) specified age as a criterion for inclusion. The severity of psychiatric symptoms for the enrolled participants ranged from mild to severe. The studies included a variety of symptom rating instruments tailored to the screening and assessment of the presence of specific psychiatric disorders, each aligned with their predominant clinical focus. Of the eleven studies (64.7%) with a primary indication of MDD, the instruments used for participant screening and inclusion were the Beck Depression Inventory [BDI] (three studies, 17.6%), Hamilton Depression Rating Scale [HAM-D] (six studies, 35.3%), and Montgomery–Åsberg Depression Rating Scale [MADRS] (one study, 5.9%). One study used the International Classification of Diseases, the 10th revision (ICD-10), to identify the presence of MDD in their enrolled participants. One study (5.9%) investigated social anxiety disorder using the Liebowitz Social Anxiety Scale (LSAS). Four studies indicated borderline personality disorder as a primary indication and employed the ICD-10 (three studies, 17.6%) and the Zanarini Rating Scale for Borderline Personality Disorder [ZAN-BPD] (one study, 5.9%) as part of the screening and clinical assessment.

*Registered Clinical Trials*. All four of the registered clinical trials provided information about the projected intention-to-treat enrollment numbers (Figure 5). Regardless of the study arm, the median total enrollment size across all trials was 31 participants, and the mean was 33 participants (*SD* = 36). Taken together, 132 participants were projected to be enrolled in these 4 clinical trials. All of the trials (100.0%) recruited both male and female participants. The mean inclusion age minimum and maximum were 29.75 (*SD* = 23.5) and 81.3 (*SD* = 17.03) years old, respectively. Of the four trials, two (50.0%) investigated MDD using the MADRS, and one trial (25.0%) investigated bipolar disorder using the MADRS as well. The remaining trial investigated social anxiety disorder (25.0%) using the LSAS.

### 2.6. Studies by Country of Origin and Funding Source

**Figure 6:**
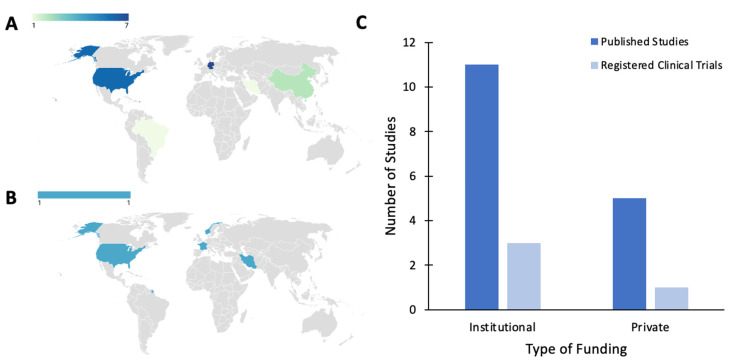
Geographical map depicting the global distribution of botulinum toxin type A clinical trials for psychiatric disorders. (**A**) Primary countries of origin for the published studies. (**B**) Primary countries of origin for the unpublished registered clinical trials. (**C**) Bar graph illustrating the funding of the included published studies and unpublished registered clinical trials.

*Published Studies*. The efficacy of BONT-A therapy for psychiatric disorders has been of research interest worldwide. Published studies have been conducted across four continents and six countries (Figure 6A). Six of the studies (35.3%) were conducted in the United States of America (USA), and the remaining research was conducted in Brazil, Switzerland, Germany, China, and Iran. Two of the studies were based both in Switzerland and Germany, and one study was based in both the USA and Germany. Sixteen out of the seventeen published studies (94.12%) indicated the type of funding received: eleven studies (68.8%) were funded by institutions (e.g., academic centers, university hospitals, etc.), and five studies (31.3%) received private funding (e.g., pharmaceutical companies, private medical centers, etc.) (Figure 6C).

*Registered Clinical Trials*. The clinical trials were conducted across three continents and four countries, spanning North America (25.0%), Asia (25.0%), and Europe (50.0%) (Figure 6B). Out of all the reviewed trials, three trials (75.0%) were funded by institutions and one trial (25.0%) received private funding (Figure 6C).

### 2.7. Studies by BONT-A Treatment Parameters

Table 1 and Table 2 list the treatment parameters and outcomes for each published study included in this systematic review, reflecting the location of BONT-A injection, dose, and used formulation. Table 3 lists the treatment parameters for each currently registered clinical trial included in the systematic review.

*Published Studies*. All 17 of the published studies (100.0%) identified the glabellar area as the site of injection, which is the area of the face involved in frowning. Of the 17 studies, 7 (41.1%) defined specific anatomical regions and muscles where the injections would take place, namely the procerus and corrugator supercilii muscles. Information about the tested BONT-A dosages was available for 94.1% of the studies. The minimum and maximum BONT-A doses tested for various indications were 20 and 100 units (U), respectively. According to the ranges provided by each trial, the average administered BONT-A dose varied between mean = 37.75 U (*SD* = 24.6 U) and mean = 44.5 U (*SD* = 23.1U) as per the anatomical variation in individuals who received treatment. Only one study [28] tested the efficacy of active BONT-A delivered at different doses (30 U vs. 50 U). Eleven trials (64.7%) provided information about the BONT-A formulation used in the trial with a corresponding manufacturer. To date, the products used in the efficacy trials of BONT-A for various psychiatric disorders include onabotulinum toxin A (ONA; Botox^®^/Vistabel^®^; Allergan Inc., Irvine, CA, USA) and incobotulinum toxin A (INCO; Xeomin^®^/Bocouture^®^, NT 201; Merz Pharmaceuticals GmbH, Frankfurt, Germany).

*Registered Clinical Trials*. Three (75.0%) out of four of the trials identified the glabellar area as the site of injection, while the remaining one trial (25.0%) did not provide any details. One trial (25.0%) specifically defined the anatomical muscles where the BONT-A or placebo saline solution was administered, listing the frown muscles (i.e., procerus and corrugator supercilii) as the targets, and one of these trials compared injections into the frown muscles and the lateral muscle orbicularis oculi involved in crow’s feet wrinkles. Information about the tested BONT-A dosage was available for three of the four (75.0%) reviewed trials. The minimum and maximum toxin doses that were tested across the psychiatric disorders were 10 U and 64 U, respectively. According to the ranges provided by each trial, the average administered BONT-A dose varied between mean = 32.7 U (*SD* = 20.03 U) and mean = 41.3 U (*SD* = 28.02 U), as per the anatomical variation in the individuals who received treatment. One trial (25.0%) provided information about the BONT-A formulation used in the trial, using onabotulinum toxin A (Allergan Inc., Irvine, CA, USA).

### 2.8. Studies by Outcome Measures

*Published Studies*. All 17 of the published studies (100.0%) defined treatment efficacy as the primary outcome, using symptom rating scales for the assessment of symptom change in various psychiatric conditions including the BDI (3 studies, 17.6%), MADRS (3 studies, 17.6%), and HAM-D (6 studies, 35.3%) for MDD, the LSAS (1 study, 5.9%) for social anxiety disorder, and the ZAN-BPD (4 studies, 23.5%) for borderline personality disorder. Eight (47.0%) of the studies defined secondary outcomes as well. These included the BDI (five studies, 29.4%) [14,15,16], Hamburg–Hannover Agitation Scale (one study, 5.9%), and HAM-D (two studies, 11.8%). Electrophysiology methods, laboratory tests, and assessment of biological rhythms were not employed in any of the trials. A study by Kruger et al. (2022) [15] used the valence inhibition task and fMRI to study the neural correlates of BONT-A therapy in borderline personality disorder, whereas Schulze et al. (2023) [31] used resting-state fMRI for the same purpose.

*Registered Clinical Trials*. Overall, all four of the trials (100%) provided information about their outcome measures. All the studies listed treatment efficacy as the primary outcome, using various instruments (LSAS, MADRS, or BDI) for evaluation. Among the trials that defined secondary outcomes, those listed included clinical and psychometric scales (two trials, 50%). Neuropsychological tests, neuroimaging, electrophysiology methods, laboratory tests, and assessment of biological rhythms were not employed in any of the trials.

### 2.9. Studies by Treatment Efficacy

*Published Studies*. Of the 11 studies addressing the potential therapeutic efficacy of BONT-A in the management of MDD, 9 of the studies (81.8%) demonstrated improvements in depressive symptoms, as evidenced by changes in scores on established scales such as the HAM-D, BDI, and MADRS (Table 1 and Table 2). The first case series on the topic by Finzi and Wasserman (2006) [16] indicated that nine out of ten subjects achieved a state of no longer being clinically depressed two months after receiving treatment with BONT-A. The other nine studies on BONT-A for MDD that followed suit all showed significant improvements in depressive symptoms post-BONT-A [25,27,28,29,30,32,33,37,38], whereas the remaining one study [24] was a reanalysis of the previously collected data [27] focused on comparing the agitation items of the HAM-D between responders and non-responders. One published study by Brin et al. (2020) [28], which investigated the impact of BONT-A for MDD at two distinct doses, namely 30 U and 50 U, observed a consistent alleviation of depressive symptoms at 30 U in comparison to placebo. This improvement was not observed at a dose of 50 U.

With regard to other psychiatric disorders beyond MDD, a case series by Finzi et al. (2018) [34] investigating bipolar depression showed that four out of six patients experienced a remission of depressive symptoms, while the remaining two displayed a notable reduction in symptoms. A case series on BONT-A treatment for social anxiety disorder by Finzi and Rosenthal (2019) [35] demonstrated a significant reduction in symptoms as assessed using LSAS. Of the four studies investigating BONT-A for borderline personality disorder (BPD) [15,26,31,36], all the studies showcased a significant reduction in borderline personality symptoms, as measured using the ZAN-BPD. In general, the studies reported few mild adverse effects associated with the BONT-A injections, including headache, muscle irritations, skin tightness, transient skin, eyelid ptosis, and dizziness.

## 3. Discussion

In the present systematic review, we performed a comprehensive search of the published studies and ongoing publicly registered clinical trials investigating the efficacy of BONT-A therapy for psychiatric disorders. We identified 17 relevant published studies and 4 unpublished trials conducted over the past 18 years, with studies being published every year since 2012. This trend highlights the general, consistent interest in conducting clinical research on BONT-A to study its therapeutic potential for the management of psychiatric disorders. The findings of the included studies support the safety and efficacy of BONT-A for the treatment of various psychiatric disorders, including MDD, bipolar disorder, social anxiety disorder, and borderline personality disorder. A total of 15 out of the 17 published studies demonstrated a reduction in symptoms as measured by the clinical outcome rating scales specific to the investigated psychiatric indications. One included study focusing on BONT-A for MDD revealed that the concurrent use of other psychotropic medications did not significantly impact the efficacy of BONT-A therapy [24]. Additionally, two of the included studies focusing on BONT-A for social anxiety and bipolar disorders reported that the participants discontinued the use of psychotropic medications due to treatment inefficacy or adverse events [34,35]. Overall, these trends suggest that BONT-A therapy holds promise as a therapeutic option for various psychiatric disorders, yielding favorable outcomes.

Moreover, six of the published studies assessed the safety of BONT-A therapy [16,25,26,27,28,32]. Four of these studies [25,26,27,28] indicated mild adverse events associated with BONT-A. The most reported adverse events across the studies included headaches, eyelid ptosis, upper respiratory tract infection, nausea, dyspepsia, and brow muscle stiffness. Less frequently reported adverse events included migraine, local skin or muscle irritation, dizziness, light sensitivity, sleep disorders, and fatigue or drowsiness. One study [26] identified six serious adverse events requiring hospitalization, attributed to the aggregation of bipolar disorder symptoms. Across the studies included in this systematic review, treatment with BONT-A was generally well-tolerated, with minimal adverse events reported among the participants. Collectively, these findings suggest a promising safety profile for the use of BONT-A in psychiatry.

The conducting of well-designed RCTs is pivotal for establishing the efficacy of BONT-A and spearheading its regulatory approval for treatment purposes, and most studies, as evidenced by our review, appear to employ a randomized, double-blind, controlled design. Our systematic review aimed to complement already available evidence from pooled [17] and meta-analyses [18,19,20,21] of BONT-A for MDD by mapping the field from a methodological standpoint and also extending the theoretical discussion for BONT-A applications to other psychiatric disorders beyond MDD; hence, we did not perform a meta-analysis due to our inherent objective to systematically map the nature of the clinical trials conducted to date and across disorders. The USA and Germany seem to be the leading countries in this research domain, as most studies are conducted and funded there. Other countries have been involved to a lesser extent, only presenting small contributions. Nevertheless, the involvement of different nations underlines the potential global interest. From an industry and manufacturing perspective, all MDD trials tested the efficacy of the manufactured onabotulinum toxin A formulation. Three borderline personality disorder studies [15,26,31] tested the efficacy of incobotulinum toxin A, although these studies were very recent. Future studies should consider investigating the therapeutic potential of other formulations of BONT-A for psychiatric disorders, including abobotulinum toxin A and incobotulinum toxin A [39].

Interestingly, while clinical trials have been successful at establishing the efficacy of BONT-A for affective disorders using a variety of clinical tools and instruments, the field is generally lacking mechanism-oriented studies with patient populations. For instance, Stark et al. (2023) [40] recently published a task-based fMRI study showing that BONT-A injections and inhibition of frowning alter the processing of emotional faces in the amygdala; these findings were confirmed in 10 healthy females. In psychiatric populations, only three fMRI studies so far have shown downregulation of the amygdala induced by BONT-A injections—notably, in MDD [13,14] and borderline personality disorder [15]. The amygdala, the key emotional brain center in humans and the central node of the emotion-to-motor transformation loop (EMTL) [7,41], has been long proposed as a transdiagnostic target for BONT-A therapy due to its involvement in maintaining negative emotions and integrating emotions [42]. At the physiological level, proprioceptive and interoceptive inputs from the muscles of facial expression feed into the EMTL, where the amygdala acts as a decision-making center that, alongside other regions in the cortex and brainstem, modulates the motor output and integrates the incoming input within the socioemotional context [7,41]. Notably, it forms a feedback loop with the anterior face area of the midcingulate cortex (area M3), and the connectivity between the two regions is responsible for the selection and production of a certain facial expression in response to a particular emotional cortex. Another relevant region participating in the EMTL is the supplementary motor area (SMA), which directly adjusts the outputs of the corticobulbar motor system for the muscles of the upper face where BONT-A is injected [7,41].

All three nodes of the EMTL (i.e., amygdala, area M3, SMA) are viable neural targets for BONT-A therapy in the context of affective disorders, and their activity and functional connectivity should be explored more in-depth after a single or a series of multiple BONT-A injections in patient populations and in a properly designed experimental setting with good controls. The field needs more mechanistic studies and clinical trials incorporating resting-state and task-based fMRI into their protocols to advance the understanding of BONT-A therapeutic mechanisms for psychiatric disorders. Electroencephalography (EEG) BONT-A studies would also be beneficial, as those are less expensive and easier to set up than fMRI experiments. EEG studies would provide invaluable information with regard to the cognitive processing associated with facial feedback inhibition due to its superb temporal resolution, and putative markers of interest may include the functional connectivity of face processing regions [43,44], frontal EEG asymmetry [45], and EEG features of emotion recognition [46] and microexpressions [47]. The selection of behavioral functional tasks with good internal validity to test the facial feedback hypothesis is also a topic for discussion; these may include voluntary facial action in response to stimuli of a certain valence (e.g., negative vs. positive) [48], mirror feedback tasks [49], facial mimicry tasks [50,51], pen-in-mouth experiments [50], and even emotional language tasks [52].

Other plausible mechanisms of BONT-A relevant to affective disorders include the upregulation of serotonin (5-HT) levels and brain-derived neurotrophic factor (BDNF) expression in the hippocampus. These theories largely stem from preclinical research [53]. Researchers conducting prospective human studies should consider performing metabolite quantification from blood samples or molecular neuroimaging, such as positron emission or single-photon emission computed tomography, to test these hypotheses in patients with affective disorders. Furthermore, in addition to the amygdala and nodes of the EMTL, other systems-level brain targets should also be explored: affective disorders are characterized by abnormal functional connectivity of major brain intrinsic connectivity networks such as the default mode, central executive, salience, sensorimotor, affective, and reward networks [54,55]. To date, it remains largely unknown what effects BONT-A injections and the subsequent inhibition of facial afferent inputs exert on the functional connectivity of these networks.

Research shows that publicly available trial results in clinical trial registries typically provide a more accurate and reliable picture of patient-relevant trial outcomes than those solely reported by corresponding medical journals [56]. The results in trial registries offer more rapid access to safety parameters and potential risks posed by investigational products, increase the accountability of investigators and responsible parties, foster adherence of trial conduct to the regulatory requirements of relevant jurisdictions, and assist in curtailing selective reporting and publication biases [57,58]. While the problem of not uploading results to trial registries is not unique to trials addressing psychiatric conditions, given the limited evidence supporting the therapeutic efficacy of BONT-A for psychiatric disorders, trial adherence to reporting requirements enhances transparency in trial conduct.

A recent meta-analysis identified five methodological concerns regarding the available evidence supporting the theoretical assumption that BONT-A ameliorates depressive symptoms [59]. Among the issues identified, the authors discussed the extraordinarily large effect sizes reported for primary outcome measures, large amounts of missing data, a preponderance of conflicts of interest, evidence of publication bias in the literature, and ineffective masking procedures considering that active BONT-A treatment, as opposed to a placebo, would induce a noticeable effect on the subjects’ appearance and the contractility of their facial musculature [59,60]. This is partially supported by our review, which revealed that 31.3% of the published studies and 25.0% of the unpublished registered trials were funded privately through pharmaceutical companies or private medical centers. As noted by Coles et al. (2019) [38], to corroborate the credibility of BONT-A efficacy for psychiatric disorders and support theoretical explanations, the field needs more institutionally funded trials led by investigators who are not affiliated with pharmaceutical companies. Routinely incorporating biomarker measures into study designs to generate and test scientific hypotheses around biological mechanisms of BONT-A on neural systems may partially mitigate this problem and make BONT-A research more academically oriented.

Additionally, new potential strategies to ensure blinding need to be explored and tested experimentally. Using saline as a placebo control would not suffice, since patients quickly note the presence or absence of the effect of the injections on their appearance [61]. If a patient observes that the received treatment clears a forehead wrinkle, they can immediately assume that BONT-A was administered, inferring that it would supposedly work for their psychiatric symptoms. Similarly, if they observe that the forehead wrinkle persists, they can assume that a placebo was administered and would carry no expectation of improvement. Using a local anesthetic agent (e.g., lidocaine) and a multiple injection protocol can potentially address this problem [61]. Similar to the way in which BONT-A induces motor paralysis, the anesthetic agent would block sensory afferents, which would permit better masking of the post-treatment physical effects [62]. A protocol with multiple injections has also been previously used in BONT-A RCTs for headaches, where the patients in the active treatment and sham arms showed similar reductions in the efficacy variables post-injection, which were attributed to the placebo effect [63].

The dosages of BONT-A used for MDD therapy fall within the ranges that have been studied extensively for 20 years (10–50 U), with a solid safety and tolerability profile [64]. To date, only one study (i.e., the 24-week phase II trial supported by Allergan Inc., Irvine, CA, USA) has involved a thorough comparison of glabellar BONT-A therapy for MDD at different doses, where the dose of 30 U was reported to have numerically superior efficacy on MADRS total scores relative to the placebo, while the dose of 50 U was not [28]. Furthermore, this trial involved female participants only, while another trial for depression in Parkinson’s disease, which did not meet the eligibility criteria for our review, specifically designated a dose of 29 U for females and a dose of 40 U for males for glabellar injections [65]. That trial was terminated due to the inability to recruit the desired sample.

Men often require higher doses than women in all treatment areas due to greater muscle mass, increased cranial size, higher density of facial blood vessels, positioning of the eyebrows, and more prominent facial rhytides [66,67]. The recommended starting dose for men is 40 U, although some men require up to 80 U depending on individual physical characteristics [66]. Underdosing remains the most common reason for the inadequate effects of BONT-A in men, which indicates that anatomical variation among participants is a crucial factor to consider when selecting the optimal dose [66]. Future trials should be able to adjust the administered BONT-A doses according to the patient’s individual anatomical characteristics. The optimal solution for trial protocols would be to specify the dosage range with predetermined minima and maxima. Based on the mean dosages revealed by our analysis, 29–40 U appears to be the optimal dose range, with the procerus and the corrugator supercilii muscles of the glabellar area as the target muscles for injection. Since only one trial has attempted to evaluate BONT-A efficacy for MDD delivered to the lateral muscle orbicularis oculi [68], more studies need to be conducted before an optimal dose can be determined for this intervention site.

Overall, we showed that all 17 of the published studies and all 4 of the registered trials that have been conducted to date have used clinical or psychometric measures as their primary or secondary outcomes, meaning that all the conclusions about the efficacy of BONT-A for psychiatric indications have been derived based on data from psychiatric interviews, self-report measures, and clinical observations. No quantitative neurobehavioral traits of MDD have been included, and the potential mechanisms of action remain largely unexplored. While the proposed effect of BONT-A for psychiatric disorders is largely rooted in the facial feedback hypothesis [14,69], this research area warrants the investigation of more biologically plausible diagnostic and treatment response markers in the form of neuropsychological tests, neuroimaging, and electrophysiology recordings, or the assessment of biological rhythms [70,71,72]. Thus, future clinical trials of BONT-A for psychiatric disorders need to incorporate quantitative neurobehavioral outcome measures and test novel hypotheses grounded in the theories of facial feedback and the biopsychosocial model of MDD as well as those of other psychiatric indications. This approach would provide insight into the mechanisms of action of BONT-A on the neurocircuitry of affect regulation, further revealing how psychiatric patients could be stratified into different types of treatments and what treatment parameters would be the most optimal for each clinical case. Furthermore, due to the transdiagnostic features shared across endophenotypes of certain disorders [23], as well as the transdiagnostic applications of interventional psychiatry treatments such as brain stimulation [73,74] or ketamine/novel psychopharmacology [75,76], the next logical step would be to examine the applications of BONT-A in the context of other psychiatric disorders with shared network-level mechanisms, notably post-traumatic stress disorder, anxiety disorders, obsessive–compulsive disorder, substance use, and eating disorders.

### Limitations

While we conducted a comprehensive systematic search across the published literature and the two largest clinical trial registries, the results of this systematic review are ultimately constrained by the extent to which the information about the studies is accurately represented and updated in these sources and databases. This might have led to an underrepresentation of older studies and trials not mandating strict adherence to registration requirements. Moreover, despite the creation of the ICRTP Registry Network in an attempt to harmonize clinical trial information originating from different international registries, trial coverage of ICRTP remains limited [77]. This review thus does not account for trials that remain unregistered or those that were registered outside of the WHO network, including those storing their information on regional or national platforms in languages other than English.

## 4. Conclusions

This methodological review of past and ongoing clinical trials evaluates the use of BONT-A injections in psychiatric conditions, either as a standalone or adjuvant treatment. It identifies prevalent research trends, underscoring both the strengths and limitations of current methodologies and shedding light on common tendencies and issues surrounding enrollment criteria, study design, BONT-A treatment parameters, and pragmatic considerations in the field. To date, most BONT-A research in psychiatry is devoted to the treatment of MDD, although studies using BONT-A for borderline personality, social anxiety, and bipolar disorders are emerging. The clinical studies overall were well-designed and executed with high rigor, and the existing efficacy data from the RCTs are encouraging. Furthermore, the administration of BONT-A appears to be highly feasible in the context of treatment for psychiatric disorders and was well-tolerated by the patients. Certain shortcomings were identified, namely the lack of studies performing neurophysiological tests, neuroimaging, electrophysiology methods, or laboratory tests, and the mechanisms of action of BONT-A for psychiatric disorders remain unexplored. While additional research needs to be conducted to ascertain the safety and efficacy of BONT-A therapy for affective disorders, this review suggests future directions toward refining research approaches in this field. By addressing the identified research gaps, the psychiatric research community can forge a more robust evidence base for BONT-A’s therapeutic potential and enhance the knowledge around the mechanism of action and therapeutic response associated with the administration of BONT-A in psychiatric populations.

## 5. Materials and Methods

This systematic review adhered to the Preferred Reporting Items for Systematic Reviews and Meta-Analyses (PRISMA) guidelines [78].

### 5.1. Search Strategy

A comprehensive search for published literature and registered unpublished clinical trials was conducted on May 4th, 2023, and the search results contained records available until May 3rd, 2023. The search for currently published literature was performed using three OVID databases (MEDLINE, Embase, APA PsycINFO), whereas the search for past and ongoing registered clinical trials was conducted using ClinicalTrials.gov (https://www.clinicaltrials.gov/; accessed on 4 May 2023) and International Clinical Trials Registry Platform (ICTRP; https://www.who.int/ictrp/en/; accessed on 4 May 2023) of the World Health Organization (WHO) public registries. The searches included combinations of the following two searchable concepts: (1) botulinum toxin* AND (2) [(*mental* OR *psychiatric* OR *psychological* OR *neuropsychiatric* OR trauma* OR *neuropsychological) adj3 (diagnos#s* OR disorder* OR disease* OR illness* OR condition*]); the second concept also included the actual names of individual psychiatric disorders, as per the Diagnostic and Statistical Manual of Mental Disorders, Fifth Edition, Text Revision (DSM-5-TR) [1]. No limits were applied to the search, and the complete search strategies are provided in Supplementary File S1. Two of the authors (I.D. and A.S.) independently performed the searches, screened the articles, and evaluated each for inclusion as per the eligibility criteria. All discrepancies were discussed and resolved through consensus with a third party (V.B.). The reference section of each included article was additionally examined.

### 5.2. Screening Process and Inclusion Criteria

Following the searches, duplicate entries from the OVID databases were identified and removed using a function of OVID. The trials were also cross-checked between the two clinical trial registries to ensure the exclusion of duplicate entries. The identified trials were screened to ensure they met the definition of a clinical trial, pertained to the therapeutic application of BONT-A injections, and enrolled human participants with a psychiatric disorder as per the DSM-5-TR, with the exception of disorders grouped into the following DSM-5-TR chapters due to their potential overlap with organic, somatic, endocrine, reproductive, or extranervous pathologies: neurodevelopmental disorders, somatic symptoms and related disorders, sleep–wake disorders, sexual dysfunctions, gender dysphoria, neurocognitive disorders, paraphilic disorders, and other mental disorders. Trials assessing two or more psychiatric comorbidities were included. Studies assessing participants with psychiatric disorders related to medical or neurological comorbidities, as well as neuropsychiatric symptoms following head injury/traumatic brain injury, childbirth (e.g., postpartum depression), stroke, or any other neurological insult, were excluded. All the participants had to receive a BONT-A injection into facial muscles for the first time. All the published studies and clinical trial entries had to be available in English, with translations of the full text permitted. There were no restrictions on the study publication or registration year, type of BONT-A formulation, or sex and gender of the participants. Pediatric studies were excluded. The publications that were excluded were those omitting the results (i.e., published protocols), animal studies, narrative or systematic reviews, conference abstracts, conference reviews, theses/dissertations, books, chapters, meta-analyses, as well as any extra publications on BONT-A therapy in psychiatric disorders that were not retrieved with English search terms. Figure 1 displays the PRISMA flow chart for this systematic review.

### 5.3. Retrieval of Published Results

Clinical trial entries that were listed as “completed” and had an associated publication were also excluded and reviewed as published studies. To gauge what proportion of the identified trials have disseminated their results, a step-wise approach was employed to identify relevant publications: (i) clinical trial entries were screened for any automatically indexed publications; (ii) the PubMed database (https://pubmed.ncbi.nlm.nih.gov/; accessed on 4 May 2023) was queried for the trial ID number using a secondary source ID (SI) field; (iii) the PubMed database was searched with a name of a principal investigator combined with the name of the trial-specified intervention or any other potentially identifying information. Two of the authors (I.D. and A.S.) independently reviewed each publication and included those reporting partial or full results of registered clinical trials (i.e., original research articles, editorials).

### 5.4. Variable Extraction and Synthesis

A database was created containing the following information captured from each study and trial entry: study publication/trial registration year, study completion and recruitment status, projected or actual enrollment numbers (intention-to-treat and per-protocol), screening and enrollment criteria (the diagnostic scale used; minimum and maximum cut-off scores; age; sex and/or gender), Phase (0–IV), comparison type (active treatment vs. placebo; patients vs. healthy controls), number of study arms, allocation (randomized vs. non-randomized), intervention model (single group, parallel, or crossover assignment), masking, and funding source (private vs. institutional). Additional variables of interest pertain to the parameters of the investigated BONT-A therapy, including the injection site and the BONT-A formulation, as well as the minimum and maximum doses (i.e., in units) of BONT-A used in that trial for any of the protocol experimental arms. The country of origin for each trial was determined as per the location of the primary responsible party (i.e., the lead center in multi-center trials). The primary and secondary outcomes for each trial were also captured to determine the types of outcome measures that were most frequently collected to assess the efficacy of BONT-A therapy in psychiatric disorders. In particular, we were interested in the use of clinical and psychometric scales, safety and feasibility metrics (e.g., adverse events, dropout rates), neuropsychological tests, neuroimaging and electrophysiology methods (e.g., magnetic resonance imaging, electroencephalography, positron emission tomography), laboratory tests (e.g., blood, saliva, urine), and assessments of biological rhythms (e.g., exercise, sleep). All the data were extracted by two independent reviewers (I.D. and A.S.), and discrepancies were resolved by a third party (V.B.). The outcomes were measured based on a descriptive univariate analysis. The descriptive statistical analysis was performed using GraphPad Prism, and data visualization was performed using Microsoft Excel and Datawrapper.

### 5.5. Quality Assessment

All the published studies included in this review were assessed for quality using the JBI (formerly Joanna Briggs Institute) Critical Appraisal Tools Checklist for use in systematic reviews [79,80] by two independent assessors (I.D. and A.S.). JBI checklists for case series [16,34,35,36,37], RCTs [15,24,25,26,27,28,29,30,31,32,33], and cohort studies [38] were used.

## Figures and Tables

**Table 1:** Treatment parameters of published studies investigating botulinum toxin injections for psychiatric disorders.

Study	Study Design	Sample Size	Allocation	Intervention Model	Masking	Drug	Site of Injection	Min Dose, U	Max Dose, U	Outcome Measures
Major Depressive Disorder										
Finzi & Wasserman (2006) [16]	Case series	10	Non-randomized	Single group	Open label	n.s.	Glabellar area	29	29	BDI
Wollmer et al. (2012) [27]	RCT	30	Randomized	Parallel	Double	Onabotulinum toxin A (Allergan Inc., Irvine, CA, USA)	Glabellar area	29	39	HAM-D, BDI, CGI
Hexsel et al. (2013) [38]	Open label	50	Non-randomized	Parallel	Open label	Onabotulinum toxin A (Allergan Inc., Irvine, CA, USA)	Glabellar area	20	20	BDI, RSE
Finzi & Rosenthal (2014) [29]	RCT	85	Randomized	Parallel	Double	Onabotulinum toxin A (Allergan Inc., Irvine, CA, USA)	Glabellar area	29	40	MADRS, treatment response and remission rates, BDI, CGI
Magid et al. (2014) [30]	RCT	30	Randomized	Crossover	Double	Onabotulinum toxin A (Allergan Inc., USA)	Glabellar area	29	39	HAM-D, BDI, PHQ-9
Wollmer et al. (2014) [24]	RCT	30	Randomized	Parallel	Double	Onabotulinum toxin A (Allergan Inc., Irvine, CA, USA)	Glabellar area	20	50	HAM-D, BDI
Zamanian et al. (2017) [32]	RCT	28	Randomized	Parallel	Double	n.s.	Glabellar area	n.s.	n.s.	BDI
Chugh et al. (2018) [37]	Open label	42	Non-randomized	Single group	Open label	Onabotulinum toxin A (Allergan Inc., Irvine, CA, USA)	Glabellar area	29	39	HAM-D, MADRS, BDI
Brin et al. (2020) [28]	RCT	255	Randomized	Parallel	Double	Onabotulinum toxin A (Allergan Inc., Irvine, CA, USA)	Glabellar area	30	50	MADRS
Zhang et al. (2021) [33]	RCT	76	Randomized	Parallel	Double	BONT-A (trade name: Hengli, Cat. No. S10970037, Lanzhou, China)	Glabellar area	100	100	HAM-D, HAMA, SDS, SAS
Li et al. (2022) [25]	RCT	120	Randomized	Parallel	Double	BONT-A (trade name: Hengli, Cat. No. S10970037, Lanzhou, China)	Glabellar area	100	100	HAM-D
**Borderline Personality Disorder**										
Kruger et al. (2016) [36]	Case series	45	Non-randomized	Single group	Open label	n.s.	Glabellar area	29	29	ZAN-BPD, BSL-23
Wollmer et al. (2022) [26]	RCT	54	Randomized	Parallel	Single group	Incobotulinum toxin A (Bocouture^®^, Merz Pharmaceuticals GmbH, Frankfurt, Germany), dissolved in 0.9% NaCl solution (100 U/2.5 mL)	Glabellar area	34	34	ZAN-BPD
Kruger et al. (2022) [15]	RCT	45	Randomized	Parallel	Single group	Incobotulinum toxin A (Bocouture^®^, Merz Pharmaceuticals GmbH, Frankfurt, Germany), dissolved in 0.9% NaCl solution (100 U/2.5 mL)	Glabellar area	34	34	ZAN-BPD, BSL-23
Schulze et al. (2023) [31]	RCT	45	Randomized	n.s.	Open label	Incobotulinum toxin A (Bocouture^®^, Merz Pharmaceuticals GmbH, Frankfurt, Germany), dissolved in 0.9% NaCl solution (100 U/2.5 mL)	Glabellar area	34	34	ZAN-BPD, BSL-23
**Social Anxiety Disorder**										
Finzi & Rosenthal (2019) [35]	Case series	6	Non-randomized	Single group	Open label	n.s.	Glabellar area	29	29	LSAS
**Bipolar Disorder**										
Finzi et al. (2018) [34]	Case series	6	Non-randomized	Single group	Open label	n.s.	Glabellar area	29	46	BDI, MADRS, QIDS-SR-16

Abbreviations: BDI = Beck Depression Inventory; BONT-A = botulinum neurotoxin type A; BSL-23 = Borderline Symptom List 23-Item; CGI = Clinical Global Impression; HAM-D = Hamilton Depression Rating Scale; HAMA = Hamilton Anxiety Scale 14-Item; LSAS = Liebowitz Social Anxiety Scale; MADRS = Montgomery–Åsberg Depression Rating Scale; n.s. = not specified; PHQ-9 = Patient Health Questionnaire-9; QIDS-SR-16 = Quick Inventory of Depressive Symptomatology Self-Report 16-Item; RCT = randomized controlled trial; RSE = Rosenberg Self-Esteem Scale; SAS = Zung Self-Rating Anxiety Scale; SDS = Zung Self-Rating Depression Scale; ZAN-BPD = Zanarini Rating Scale for Borderline Personality Disorder.

**Table 2:** Results of published studies investigating botulinum toxin injections for psychiatric disorders.

Study	Study Design	Sample Size	Outcome	Pre-Treatment Mean Score (Active Arm)	Post-Treatment Mean Score (Active Arm)	Results
Major Depressive Disorder						
Finzi & Wasserman (2006) [16]	Case series	10	BDI-II	30.7	8.1	9 of 10 clinically depressed individuals were no longer depressed 2 months after treatment
Wollmer et al. (2012) [27]	RCT	30	HAM-D	21.4	11.33	6 weeks post-treatment, HAM-D scores were reduced by 47.1% for the BONT-A group compared to 9.2% for the placebo group Treatment-dependent clinical improvement was also reflected in BDI and CGI scores
Hexsel et al. (2013) [38]	Open label	50	BDI	27.4	12.5	Significant improvement in depression symptoms and self-esteem Maximum effect occurred within first 8 weeks after treatment
Finzi & Rosenthal (2014) [29]	RCT	85	MADRS	31.6	16.9	At 6 weeks post-injection, response rates were 52% for BONT-A, 15% for placebo Remission rate was 27% with BONT-A and 7% with placebo MADRS scores were reduced by 47% for BONT-A and 21% for placebo
Magid et al. (2014) [30]	RCT	30	HAM-D	27.9 (BONT-A at week 0) vs. 23.7 (BONT-A at week 12)	15.2 (BONT-A at week 0) vs. 15.3 (BONT-A at week 12)	Patients who received BONT-A had a significant reduction in depressive symptoms compared to patients who received the placebo Improvement in depressive symptoms continued over 24 weeks, even though the cosmetic effects of BONT-A wore off at 12–16 weeks
Wollmer et al. (2014) [24]	RCT	30	HAMD	1.07	0.67	Responders had significantly higher HAM-D item 9 (agitation) scores at baseline [1.56 + 0.88 vs. 0.33 + 0.52, t(13) = 3.04, d = 1.7, *p* = 0.01], while no other single item of the HAM-D or BDI was associated with treatment response. The agitation score had an overall precision of 78% in predicting the response in a receiver operating characteristic analysis (area under the curve, AUC = 0.87)
Zamanian et al. (2017) [32]	RCT	28	BDI	30.86	19	Statistically significant difference in BDI scores between active and placebo groups at week 6
Chugh et al. (2018) [37]	Open label	42	HAMD	32.8	23.9	Almost all patients improved clinically, with depression scores dropping by 27% Treatment effects did not differ between male and female patients
Brin et al. (2020) [28]	RCT	255	MADRS	32	17.2	30 U showed consistent improvement in depressive symptoms compared to placebo up to week 15. Treatment with 50 U did not improve depressive symptoms compared to placebo; may be partially attributed to high placebo response
Zhang et al. (2021) [33]	RCT	76	HAMD	14.04	4.89	Scores of HAM-D, HAMA, SDS, and SAS decreased significantly in both BONT-A and sertraline groups after treatment for 12 weeks. Overall, there were no differences in decreased magnitude between the two groups (*p* > 0.05). The HAMA, SDS, and SAS results showed that the onset time of BONT-A was earlier than that of sertraline. Side effects rates were 15.38% for BONT-A and 33.33% for sertraline
Li et al. (2022) [25]	RCT	120	HAMD	12.82	5.78	There was a significant improvement in depressive symptoms of the BONT-A group compared to the placebo group throughout the 12-week follow-up period
**Borderline Personality Disorder**						
Kruger et al. (2016) [36]	Case series	45	ZAN-BPD	17.67	4.67	2–6 weeks following the injection of BONT-A, the symptoms of borderline personality disorder as measured by ZAN-BPD and BSL-23 had improved by 49–94% from baseline values (Wilcoxon signed ranks test, *p* < 0.05)
Wollmer et al. (2022) [26]	RCT	54	ZAN-BPD	15.41	n.s.	Participants showed significant improvements at the primary efficacy end time point in both treatment groups (BONT-A: M = –0.39, SD = 0.39; ACU: M = –0.35, SD = 0.42), but no superior effect of the BONT-A condition in comparison with ACU was found—F(1, 5323) = 0.017, *p* = 0.68)
Kruger et al. (2022) [15]	RCT	45	ZAN-BPD	16.04	10.35	Borderline personality disorder symptoms significantly decreased over time by 6.11 points (BONT-A 5.67, 2 ACU 6.62) in both groups, as assessed by expert rating using the ZAN-BPD scale (F(1, 42) = 44.71, *p* < 0.001, η = 0.51). BSL-23 scores displayed a similar pattern over time, with an average decrease of 0.58 points (BONT-A 0.58, ACU 0.58) (F(1, 41) = 20.24, *p* < 0.001, η^2^ = 0.33) but no significant differences between the groups (F(1, 41) = 0.86, *p* = n.s.) and no interaction effects (F(1, 41) = 0.000, *p* = n.s.)
Schulze et al. (2023) [31]	RCT	45	ZAN-BPD	16.04	10.35	After 4 weeks, both groups showed a reduction in borderline symptoms. However, the anterior cingulate cortex (ACC) and the face area in the primary motor cortex (M1) displayed aberrant rsFC after BONT-A compared to ACU treatment. The M1 area showed higher rsFC to the ACC after BONT-A treatment compared to ACU treatment. In addition, the ACC displayed an increased rsFC to the M1 area as well as a decreased rsFC to the right cerebellum
**Social Anxiety Disorder**						
Finzi & Rosenthal (2019) [35]	Case series	6	BDI	n.s.	n.s.	8–12 weeks after BONT-A injections, LSAS scores in individual patients decreased from baseline values, and the range of reduction was 24–100% (Wilcoxon signed rank test, *p* ≤ 0.05)
**Bipolar Disorder**						
Finzi et al. (2018) [34]	Case series	6	BDI, QIDS-SR-16, MADRS	n.s.	n.s.	4 of 6 patients with bipolar depression experienced a remission following treatment with BONT-A, and the other 2 patients experienced a reduction in depressive symptoms When the effect of BONT-A on the frown muscles began to wear off, depressive symptoms returned and retreatment with BONT-A provided successful relief of depressive symptoms again

Abbreviations: ACU = acupuncture (control condition); BDI = Beck Depression Inventory; BONT-A = botulinum neurotoxin type A; BSL-23 = Borderline Symptom List 23-Item; CGI = Clinical Global Impression; HAM-D = Hamilton Depression Rating Scale; HAMA = Hamilton Anxiety Scale 14-Item; LSAS = Liebowitz Social Anxiety Scale; MADRS = Montgomery–Åsberg Depression Rating Scale; n.s. = not specified; QIDS-SR-16 = Quick Inventory of Depressive Symptomatology Self-Report 16-Item; RCT = randomized controlled trial; rsFC = resting-state functional connectivity; SAS = Zung Self-Rating Anxiety Scale; SDS = Zung Self-Rating Depression Scale; ZAN-BPD = Zanarini Rating Scale for Borderline Personality Disorder.

**Table 3:** Treatment parameters and outcomes of registered clinical trials investigating botulinum toxin injections for psychiatric disorders.

Clinical Trial	Phase	Study Design	Sample Size	Allocation	Intervention Model	Masking	Drug	Site of Injection	Min Dose, U	Max Dose, U	Clinical Indication	Primary Outcomes	Secondary Outcomes
NCT03833063	I	RCT	0	Randomized	Crossover	Quadruple	n.s.	Glabellar area	n.s.	n.s.	MDD	Response (MADRS)	Remission (MADRS); GDS; QOL-AD; safety/tolerability
NCT03484754	n.s.	RCT	58	Randomized	Parallel	Open label	Onabotulinum toxin A (Allergan Inc., Irvine, CA, USA)	Glabellar area (corrugator, procerus) vs. crow’s feet area (orbicularis oculi)	10	10	MDD	MADRS (proportion of patients with improvements in depressive symptoms)	N/A
NCT03078270	n.s.	RCT	4	Randomized	Parallel	Double	Clostridium botulinum type A neurotoxin complex; 0.5 mg Albumin Human; 0.9 mg NaCl	n.s.	40	50	Social anxiety disorder	LSAS	BDI
IRCT20160523028008N7	II–III	RCT	70	Randomized	Parallel	Double	n.s.	Glabellar area	48	64	Bipolar disorder	HAM-D	N/A

Abbreviations: GDS = Geriatric Depression Scale; HAM-D = Hamilton Depression Rating Scale; LSAS = Liebowitz Social Anxiety Scale; MADRS = Montgomery–Åsberg Depression Rating Scale; MDD = major depressive disorder; n.s. = not specified; N/A = not applicable; QOL-AD = Quality of Life-Alzheimer’s Disease; RCT = randomized controlled trial; U = units.

## Data Availability

The data supporting this systematic review are from previously reported studies and clinical trial entries (ClinicalTrials.gov, ICTRP), which have been cited. The processed data generated and analyzed during this systematic review are available upon request from the corresponding author.

## Supplementary Materials

The following supporting information can be downloaded at: https://www.mdpi.com/article/10.3390/toxins16040191/s1, File S1: Literature Search Strategy for the Systematic Review; File S2: Eligibility Criteria for the Systematic Review; File S3: Tables S1–S3 Results of the Quality Assessment; File S4: PRISMA Checklists.
