# Supplementary material for: Botulinum Toxin Injections for Psychiatric Disorders: A Systematic Review of the Clinical Trial Landscape
Source: Toxins (Basel). 2024 Apr 15;16(4):191. doi: 10.3390/toxins16040191 (PMC11054929; doi:10.3390/toxins16040191)
Supplement: Supplementary file 1 [file toxins-16-00191-s001.zip › toxins-2893137-supplementary_final/Supplementary Materials - revision/Supplementary - File 2 - Eligibility Criteria.docx]

**Eligibility criteria**

***Inclusion criteria***

- Original published research studies (journal articles);
- Case reports, case series;
- Letters, editorials, notes, preprints, errata/corrigenda, conference original research papers, protocol papers, unpublished clinical trials from ClinicalTrials.gov and WHO ICTRP;
- If a clinical trial has an associated publication, it is reviewed as a published study and NOT included in the unpublished clinical trials review (ie, removed from the total count);
- Protocol papers with no results are reviewed as clinical trials and not as published studies;
- At least one of the study groups must have a psychiatric disorder; these were defined as per APA DSM-5. Excluded disorder categories: *neurodevelopmental disorders (except for ADHD and autism spectrum – these are included), somatic disorders, sleep-wake disorders, breathing-related sleep disorders, parasomnias, sexual dysfunction, gender dysphoria, paraphilic disorders*, *neurocognitive disorders*;
- Studies administered botulinum neurotoxin type A (BONT-A) injection into the facial muscles. At least 1 arm should have received verum BONT-A;
- BONT-A in combination with other treatments (e.g., BONT-A + medications; BONT-A + psychotherapy) are allowed for inclusion;
- If BONT-A + some other treatment (e.g., BONT-A followed by physiotherapy) as part of the same arm, studies are allowed for inclusion;
- Participants with two or more psychiatric comorbidities allowed for inclusion (e.g., depression + anxiety; depression + substance use), as long as it is one of the DSM-5 disorders that are part of our inclusion criteria;
- BONT-A should be administered as an intervention in a clinical study setting (no basic research studies). A single injection of BONT-A is allowed for inclusion;
- Participants aged 18+ (geriatric studies are allowed for inclusion, and there is no upper age limit);
- English language;

***Exclusion criteria***

- Reviews, systematic reviews, books, chapters, theses/dissertations, conference abstracts, conference reviews, meta-analyses;
- Excluded if BONT-A is assessed for the following neuropsychiatric manifestations: *neurodevelopmental disorders (except for autism and ADHD – these are included), somatic disorders, sleep-wake disorders, breathing-related sleep disorders, parasomnias, sexual dysfunction, gender dysphoria, paraphilic disorders, neurocognitive disorders (e.g., Huntington’s, Parkinson’s, Lewy body)*;
- No neurodegenerative/neurocognitive disorders (ie, dementias);
- No restriction on the type of BONT-A formulation;
- Participants aged <18 (pediatric studies);
- Participants with psychiatric disorders in the context of medical comorbidities (e.g., cancer) or neurological comorbidities (e.g., pain, epilepsy);
- Participants with neuropsychiatric symptoms following head injury/traumatic brain injury, childbirth (postpartum depression), stroke, or any other neurological insult;
- Non-English language;
- Studies on animal models;
- Electrophysiological studies measuring the effect of BONT-A on the function of neurons (without a clear report of human patient outcomes);
- Studies of BONT-A on basic human neurobehavioural functions (e.g., emotion regulation, memory, executive control) without a clear underlying psychiatric disorder that’s part of our criteria (e.g., effect of BONT-A on fear conditioning in humans/healthy people; effect of BONT-A on memory and language learning in humans/healthy people).
