# Supplementary material for: Botulinum Toxin Injections for Psychiatric Disorders: A Systematic Review of the Clinical Trial Landscape
Source: Toxins (Basel). 2024 Apr 15;16(4):191. doi: 10.3390/toxins16040191 (PMC11054929; doi:10.3390/toxins16040191)
Supplement: Supplementary file 1 [file toxins-16-00191-s001.zip › toxins-2893137-supplementary_final/Supplementary Materials - revision/Supplementary - File 1 - Search Strategy.docx]

**Search Strategy: Published Studies (OVID)**

Database(s): **Embase Classic+Embase**1947 to 2023 May 03**, APA PsycInfo**1806 to April Week 4 2023**, Ovid MEDLINE(R) ALL**1946 to May 03, 2023 
Search Strategy:

| **#** | **Searches** | **Results** |
| --- | --- | --- |
| 1 | exp Botulinum Toxins/ or exp Botulinum Toxin/ | 41215 |
| 2 | botulinum toxin*.tw,id,kf. | 43007 |
| 3 | botulinum neurotoxin*.tw,id,kf. | 9057 |
| 4 | (bont-a or bont-b or btx or botulin or botulinum).tw,id,kf. | 61198 |
| 5 | (onabotulin* or abobotulin* or incobotulin* or rimabotulin*).tw,id,kf. | 8012 |
| 6 | (botox or dysport or jeuveau or xeomin or vistabel or daxxify or botulax or bocouture or myobloc).tw,id,kf. | 13333 |
| 7 | or/1-6 | 77852 |
| 8 | exp Mental Disorders/ | 5219589 |
| 9 | ((mental or psychiatric or psychological or neuropsychiatric or trauma* or neuropsychological) adj3 (diagnos#s or disorder* or disease* or illness* or condition*)).tw,id,kf. | 844303 |
| 10 | (anxiety or agoraphobia or phobia or panic or phobic).tw,id,kf. | 915128 |
| 11 | (posttraumatic stress disorder* or post traumatic stress disorder* or post-traumatic stress disorder* or PTSD).tw,id,kf. | 155424 |
| 12 | (obsessive compulsive disorder* or obsessive-compulsive disorder* or OCD).tw,id,kf. | 64979 |
| 13 | (binge eating disorder* or binge-eating disorder* or anorexi* or bulimi* or orthorexi* or pica or feeding disorder* or eating disorder*).tw,id,kf. | 195453 |
| 14 | (bipolarity or mania or hypomania or manic or hypomanic or cyclothymic or cyclothymia).tw,id,kf. | 81339 |
| 15 | ((bipolar or mood or affect* or depressive) adj3 (disorder* or illness*)).tw,id,kf. | 472289 |
| 16 | (major depressi* or dysthymi*).tw,id,kf. | 199811 |
| 17 | (MDD or TRD).tw,id,kf. | 65623 |
| 18 | ((treatment resistant or treatment-resistant) adj2 (depression or bipolar)).tw,id,kf. | 13490 |
| 19 | (ADHD or attention deficit hyperactivity disorder*).tw,id,kf. | 133728 |
| 20 | (autism or asperger*).tw,id,kf. | 203825 |
| 21 | (conduct disorder* or defiant disorder* or gambling disorder* or pathological gambling).tw,id,kf. | 36144 |
| 22 | (trichotillomania* or hair pulling disorder* or hair-pulling disorder*).tw,id,kf. | 4753 |
| 23 | (psychopath* or sociopath*).tw,id,kf. | 224236 |
| 24 | ((borderline or antisocial or histrionic or schizoid or schizotypal or paranoi* or delusion* or compulsive or obsessive-compulsive or passive-aggressive or avoidant or dependent) adj3 (disorder* or illness*)).tw,id,kf. | 121640 |
| 25 | (schizophreni* or psychos#s or psychotic or hallucination* or delusion* or schizotyp*).tw,id,kf. | 710052 |
| 26 | exp Substance-Related Disorders/ or exp "substance use disorder"/ or exp addiction/ | 824016 |
| 27 | ((substance or drug or alcohol or tobacco or caffeine or opioid or cocaine or amphetamine or cannabis or marijuana or nicotine) adj3 (dependence or abuse or addiction* or disorder*)).tw,id,kf. | 503940 |
| 28 | or/8-27 | 6416933 |
| 29 | 7 and 28 | 5999 |
| 30 | remove duplicates from 29 | 5146 |

**Search Strategy: Unpublished Clinical Trials (ClinicalTrials.gov, WHO ICTRP)**

**Clinical Trials.gov**

Number of searches: 22

| **Condition or Disease** | **Condition or Disease Search Terms** | **Intervention/Treatment**  **Search Terms** | **# CTs Returned** |
| --- | --- | --- | --- |
| General | (mental disorder OR psychiatric disorder OR psychological disorder OR neuropsychiatric disorder OR trauma disorder OR neuropsychological disorder OR mental disease OR psychiatric disease OR psychological disease) | **(**botulinum toxin OR botulinum neurotoxin OR bont-a OR bont-b OR btx OR botulin OR onabotulin OR abobotulin OR incobotulin OR rimabotulin OR botox OR dysport OR jeuveau OR xeomin OR vistabel OR daxxify OR botulax OR bocouture OR myobloc) | 124 |
| General (2) | (neuropsychiatric disease OR trauma disease OR neuropsychological disease OR mental diagnosis OR psychiatric diagnosis OR psychological diagnosis OR neuropsychiatric diagnosis OR trauma diagnosis OR neuropsychological diagnosis OR mental condition) | **(**botulinum toxin OR botulinum neurotoxin OR bont-a OR bont-b OR btx OR botulin OR onabotulin OR abobotulin OR incobotulin OR rimabotulin OR botox OR dysport OR jeuveau OR xeomin OR vistabel OR daxxify OR botulax OR bocouture OR myobloc) | 79 |
| General (3) | (psychiatric condition OR psychological condition OR neuropsychiatric condition OR trauma condition OR neuropsychological condition OR mental illness OR psychiatric illness) | **(**botulinum toxin OR botulinum neurotoxin OR bont-a OR bont-b OR btx OR botulin OR onabotulin OR abobotulin OR incobotulin OR rimabotulin OR botox OR dysport OR jeuveau OR xeomin OR vistabel OR daxxify OR botulax OR bocouture OR myobloc) | 134 |
| General (4) | (psychological illness OR neuropsychiatric illness OR trauma illness OR neuropsychological illness OR mental health) | **(**botulinum toxin OR botulinum neurotoxin OR bont-a OR bont-b OR btx OR botulin OR onabotulin OR abobotulin OR incobotulin OR rimabotulin OR botox OR dysport OR jeuveau OR xeomin OR vistabel OR daxxify OR botulax OR bocouture OR myobloc) | 0 |
| Eating Disorder | (anorexia nervosa OR binge-eating OR purging OR avoidant/restrictive food intake disorder OR pica OR ARFID OR eating disorder) | **(**botulinum toxin OR botulinum neurotoxin OR bont-a OR bont-b OR btx OR botulin OR onabotulin OR abobotulin OR incobotulin OR rimabotulin OR botox OR dysport OR jeuveau OR xeomin OR vistabel OR daxxify OR botulax OR bocouture OR myobloc) | 1 |
| Bipolar Disorder | (bipolar disorder OR bipolar illness OR BPI OR BPII OR BD-I OR BD-II OR treatment resistant bipolar disorder OR hypomania OR mania OR manic depression OR manic-depression OR MDI OR bipolar depression) | **(**botulinum toxin OR botulinum neurotoxin OR bont-a OR bont-b OR btx OR botulin OR onabotulin OR abobotulin OR incobotulin OR rimabotulin OR botox OR dysport OR jeuveau OR xeomin OR vistabel OR daxxify OR botulax OR bocouture OR myobloc) | 0 |
| Mood Disorders (not BD/MDD) | (cyclothymic disorder OR cyclothymia OR affective disorder OR affective illness OR seasonal affect disorder OR SAD) | **(**botulinum toxin OR botulinum neurotoxin OR bont-a OR bont-b OR btx OR botulin OR onabotulin OR abobotulin OR incobotulin OR rimabotulin OR botox OR dysport OR jeuveau OR xeomin OR vistabel OR daxxify OR botulax OR bocouture OR myobloc) | 8 |
| Depression | (depression OR major depressive disorder OR MDD OR treatment resistant depression OR dysthymia OR disruptive mood dysregulation disorder OR DMDD OR premenstrual dysphoric disorder OR PMDD OR persistent depressive disorder OR PDD OR dysthymia) | **(**botulinum toxin OR botulinum neurotoxin OR bont-a OR bont-b OR btx OR botulin OR onabotulin OR abobotulin OR incobotulin OR rimabotulin OR botox OR dysport OR jeuveau OR xeomin OR vistabel OR daxxify OR botulax OR bocouture OR myobloc) | 10 |
| Obsessive-compulsive and Related Disorders | (obsessive-compulsive disorder OR OCD OR obsession OR compulsion OR body dysmorphic disorder OR BDD) | **(**botulinum toxin OR botulinum neurotoxin OR bont-a OR bont-b OR btx OR botulin OR onabotulin OR abobotulin OR incobotulin OR rimabotulin OR botox OR dysport OR jeuveau OR xeomin OR vistabel OR daxxify OR botulax OR bocouture OR myobloc) | 0 |
| Obsessive-compulsive and Related Disorders (2) | (hoarding disorder OR trichotillomania OR excoriation OR olfactory reference disorder OR olfactory reference syndrome OR ORS OR hypochondriasis OR body-focused repetitive behavior disorder OR BFRBDs) | **(**botulinum toxin OR botulinum neurotoxin OR bont-a OR bont-b OR btx OR botulin OR onabotulin OR abobotulin OR incobotulin OR rimabotulin OR botox OR dysport OR jeuveau OR xeomin OR vistabel OR daxxify OR botulax OR bocouture OR myobloc) | 0 |
| Psychotic Disorders | (schizophrenia OR schizoaffective disorder OR SZA OR non-affective psychosis OR schizotypal or schizoid OR psychosis OR delusional disorder OR delusion OR hallucination OR cataonia OR schizophreniform disorder) | **(**botulinum toxin OR botulinum neurotoxin OR bont-a OR bont-b OR btx OR botulin OR onabotulin OR abobotulin OR incobotulin OR rimabotulin OR botox OR dysport OR jeuveau OR xeomin OR vistabel OR daxxify OR botulax OR bocouture OR myobloc) | 84 |
| Autism Spectrum Disorders | (autism OR asperger) | **(**botulinum toxin OR botulinum neurotoxin OR bont-a OR bont-b OR btx OR botulin OR onabotulin OR abobotulin OR incobotulin OR rimabotulin OR botox OR dysport OR jeuveau OR xeomin OR vistabel OR daxxify OR botulax OR bocouture OR myobloc) | 0 |
| Substance Use Disorders | (addiction OR drug dependence OR drug abuse OR substance use OR substance abuse OR drug misuse OR drug use OR substance use disorder OR alcohol use disorder OR substance-related disorder OR intoxication OR drug withdrawal) | **(**botulinum toxin OR botulinum neurotoxin OR bont-a OR bont-b OR btx OR botulin OR onabotulin OR abobotulin OR incobotulin OR rimabotulin OR botox OR dysport OR jeuveau OR xeomin OR vistabel OR daxxify OR botulax OR bocouture OR myobloc) | 5 |
| Substance Use Disorders (2) | (alcohol addiction OR tobacco addiction OR caffeine addiction OR opioid addiction OR amphetamine addiction OR cannabis addiction OR marijuana addiction OR nicotine addiction OR alcohol dependence OR tobacco dependence OR caffeine dependence) | **(**botulinum toxin OR botulinum neurotoxin OR bont-a OR bont-b OR btx OR botulin OR onabotulin OR abobotulin OR incobotulin OR rimabotulin OR botox OR dysport OR jeuveau OR xeomin OR vistabel OR daxxify OR botulax OR bocouture OR myobloc) | 0 |
| Substance Use Disorders (3) | (amphetamine dependence OR cannabis dependence OR marijuana dependence OR nicotine dependence OR alcohol abuse OR tobacco abuse OR caffeine abuse OR opioid abuse OR amphetamine abuse OR cannabis abuse OR marijuana abuse OR nicotine abuse) | **(**botulinum toxin OR botulinum neurotoxin OR bont-a OR bont-b OR btx OR botulin OR onabotulin OR abobotulin OR incobotulin OR rimabotulin OR botox OR dysport OR jeuveau OR xeomin OR vistabel OR daxxify OR botulax OR bocouture OR myobloc) | 0 |
| Posttraumatic Stress Disorder | (posttraumatic stress disorder OR post traumatic stress disorder or post-traumatic stress disorder OR PTSD) | **(**botulinum toxin OR botulinum neurotoxin OR bont-a OR bont-b OR btx OR botulin OR onabotulin OR abobotulin OR incobotulin OR rimabotulin OR botox OR dysport OR jeuveau OR xeomin OR vistabel OR daxxify OR botulax OR bocouture OR myobloc) | 0 |
| Personality Disorders | (borderline disorder OR antisocial disorder OR histrionic disorder OR schizoid OR schizotypal disorder OR paranoid OR paranoia OR delusional OR compulsive OR obsessive-compulsive disorder OR passive-aggressive disorder OR avoidant OR dependent) | **(**botulinum toxin OR botulinum neurotoxin OR bont-a OR bont-b OR btx OR botulin OR onabotulin OR abobotulin OR incobotulin OR rimabotulin OR botox OR dysport OR jeuveau OR xeomin OR vistabel OR daxxify OR botulax OR bocouture OR myobloc) | 2 |
| Attention Deficit Disorders | (ADHD OR attention deficit hyperactivity disorder) | **(**botulinum toxin OR botulinum neurotoxin OR bont-a OR bont-b OR btx OR botulin OR onabotulin OR abobotulin OR incobotulin OR rimabotulin OR botox OR dysport OR jeuveau OR xeomin OR vistabel OR daxxify OR botulax OR bocouture OR myobloc) | 0 |
| Psychopathy/Sociopathy | (psychopathy OR sociopathy) | **(**botulinum toxin OR botulinum neurotoxin OR bont-a OR bont-b OR btx OR botulin OR onabotulin OR abobotulin OR incobotulin OR rimabotulin OR botox OR dysport OR jeuveau OR xeomin OR vistabel OR daxxify OR botulax OR bocouture OR myobloc) | 0 |
| Gambling Disorders | (conduct disorder OR defiant disorder OR gambling disorder OR pathological gambling) | **(**botulinum toxin OR botulinum neurotoxin OR bont-a OR bont-b OR btx OR botulin OR onabotulin OR abobotulin OR incobotulin OR rimabotulin OR botox OR dysport OR jeuveau OR xeomin OR vistabel OR daxxify OR botulax OR bocouture OR myobloc) | 10 |
| Anxiety Disorders | (anxiety OR generalized anxiety disorder OR agoraphobia OR phobia OR panic OR phobic) | **(**botulinum toxin OR botulinum neurotoxin OR bont-a OR bont-b OR btx OR botulin OR onabotulin OR abobotulin OR incobotulin OR rimabotulin OR botox OR dysport OR jeuveau OR xeomin OR vistabel OR daxxify OR botulax OR bocouture OR myobloc) | 7 |
| Hair Pulling Disorders | (trichotillomania OR hair pulling disorder OR hair-pulling disorder) | **(**botulinum toxin OR botulinum neurotoxin OR bont-a OR bont-b OR btx OR botulin OR onabotulin OR abobotulin OR incobotulin OR rimabotulin OR botox OR dysport OR jeuveau OR xeomin OR vistabel OR daxxify OR botulax OR bocouture OR myobloc) | 0 |

**WHO ICTRP**

**Note:** It appears that doing a general search for Botox (+variations) captures the most trials that could be relevant.

Number of searches: 1

| **Condition or Disease** | **Condition or Disease Search Terms** | **Intervention/Treatment**  **Search Terms** | **# CTs Returned** |
| --- | --- | --- | --- |
| Unspecified | Unspecified | **(**botulinum toxin OR botulinum neurotoxin OR bont-a OR bont-b OR btx OR botulin OR onabotulin OR abobotulin OR incobotulin OR rimabotulin OR botox OR dysport OR jeuveau OR xeomin OR vistabel OR daxxify OR botulax OR bocouture OR myobloc) | 1777 |
