# Supplementary material for: Botulinum Toxin Injections for Psychiatric Disorders: A Systematic Review of the Clinical Trial Landscape
Source: Toxins (Basel). 2024 Apr 15;16(4):191. doi: 10.3390/toxins16040191 (PMC11054929; doi:10.3390/toxins16040191)
Supplement: Supplementary file 1 [file toxins-16-00191-s001.zip › toxins-2893137-supplementary_final/Supplementary Materials - revision/Supplementary - File 3 - Tables S1-3.docx]

**Quality Assessment of included published studies using the JBI Critical Appraisal Tools Checklist for Use in Systematic Reviews**

Reference**:** Tufanaru, C; Munn, Z; Aromataris, E; Campbell, J; Hopp, L Chapter 3: Systematic Reviews of Effectiveness. In *JBI Manual for Evidence Synthesis*; JBI, 2020 ISBN 978-0-648-84880-6.

**Table S1**. Quality assessment of randomized controlled trials included in the systematic review

**Colour Coding: Yes No Unclear N/A**

|  |  |  |  |  |  |  |  |  |  |  |  |
| --- | --- | --- | --- | --- | --- | --- | --- | --- | --- | --- | --- |
| **Study** | | **Q1** | **Q2** | **Q3** | **Q4** | **Q5** | **Q6** | **Q7** | **Q8** | **Q9** | **Q10** |
| Finzi et al., 2018 | | No | Yes | Unclear | Yes | No | No | No | Yes | No | Unclear |
| Finzi & Rosenthal, 2019 | | No | Yes | Yes | No | No | No | No | Yes | No | Yes |
| Finzi & Wasserman, 2006 | | Yes | Yes | Yes | No | Unclear | No | Yes | Yes | No | Yes |
| Kruger et al., 2016 | | No | Yes | Unclear | Yes | Unclear | No | No | Yes | No | Yes |
| Chugh et al., 2018 | | Unclear | Yes | Yes | Yes | No | No | No | Yes | No | Yes |

**Q1**: Were there clear criteria for inclusion in the case series? **Q2**: Was the condition measured in a standard, reliable way for all participants included in the case series? **Q3**: Were valid methods used for identification of the condition for all participants included in the case series? **Q4**: Did the case series have consecutive inclusion of participants? **Q5**: Did the case series have complete inclusion of participants? **Q6**: Was there clear reporting of the demographics of the participants in the study? **Q7**: Was there clear reporting of clinical information of the participants? **Q8**: Were the outcomes or follow up results of cases clearly reported? **Q9**: Was there clear reporting of the presenting site(s)/clinic(s) demographic information? **Q10**: ​​Was statistical analysis appropriate?

| **Table S2.** Quality assessment of randomized controlled trials included in the systematic review | | | | | | | | | | | | | |
| --- | --- | --- | --- | --- | --- | --- | --- | --- | --- | --- | --- | --- | --- |
| **Study** | **Q1** | **Q2** | **Q3** | **Q4** | **Q5** | **Q6** | **Q7** | **Q8** | **Q9** | **Q10** | **Q11** | **Q12** | **Q13** |
| Wollmer et al., 2014 | Yes | Yes | Yes | Yes | Yes | Yes | Yes | Yes | Yes | Unclear | Yes | Yes | Yes |
| Li et al., 2022 | Yes | Yes | Yes | Yes | Yes | Yes | Yes | Yes | Yes | Yes | Yes | Yes | Yes |
| Wollmer et al., 2022 | Yes | No | Yes | No | No | Yes | Yes | Yes | Yes | Yes | Yes | Yes | Yes |
| Wollmer et al., 2012 | Yes | Yes | No | Yes | Yes | Yes | Yes | Yes | Yes | Yes | Yes | Yes | Yes |
| Brin et al., 2020 | Yes | Yes | Yes | Yes | Yes | Yes | Yes | Yes | Yes | Yes | Yes | Yes | Yes |
| Finzi & Rosenthal, 2014 | Yes | Yes | Yes | Yes | Yes | Yes | Yes | Yes | Yes | Unclear | Yes | Yes | Yes |
| Magid et al., 2014 | Yes | Yes | Yes | Yes | Yes | Yes | Yes | Yes | Yes | Unclear | Yes | Yes | Yes |
| Schulze et al., 2023 | Yes | No | Unclear | No | No | Yes | Yes | Yes | Yes | Yes | Yes | Yes | Yes |
| Zamanian et al., 2017 | Yes | Yes | Yes | Yes | Yes | Yes | Yes | Yes | Yes | Yes | Yes | Yes | Yes |
| Kruger et al., 2022 | Yes | No | Unclear | No | No | Yes | Yes | Yes | Yes | Yes | Yes | Yes | Yes |
| Zhang et al., 2021 | Yes | Yes | Yes | Yes | Yes | Yes | Yes | Yes | Yes | Yes | Yes | Yes | Yes |

**Q1**: Was true randomization used for assignment of participants to treatment groups? **Q2**: Was allocation to treatment groups concealed? **Q3**: Were treatment groups similar at the baseline? **Q4**: Were participants blind to treatment assignment? **Q5**: Were those delivering treatment blind to treatment assignment? **Q6**: Were treatment groups treated identically other than the intervention of interest? **Q7**: Were outcomes assessors blind to treatment assignment? **Q8**: Were outcomes measured in the same way for treatment groups? **Q9**: Were outcomes measured in a reliable way? **Q10**: Was follow up complete and if not, were differences between groups in terms of their follow up adequately described and analysed? **Q11**: Were participants analysed in the groups to which they were randomized? **Q12**: Was appropriate statistical analysis used? **Q13**: Was the trial design appropriate, and any deviations from the standard RCT design (individual randomization, parallel groups) accounted for in the conduct and analysis of the trial?

| **Table S3**. Quality assessment of cohort studies included in the systematic review | | | | | | | | | | | |
| --- | --- | --- | --- | --- | --- | --- | --- | --- | --- | --- | --- |
| **Study** | **Q1** | **Q2** | **Q3** | **Q4** | **Q5** | **Q6** | **Q7** | **Q8** | **Q9** | **Q10** | **Q11** |
| Hexsel et al., 2013 | Yes | Yes | Yes | Yes | Unclear | Yes | Yes | Yes | Yes | Unclear | Yes |

**Q1**: Were the two groups similar and recruited from the same population? **Q2**: Were the exposures measured similarly to assign people to both exposed and unexposed groups? **Q3**: Was the exposure measured in a valid and reliable way? **Q4**: Were confounding factors identified? **Q5**: Were strategies to deal with confounding factors stated? **Q6**: Were the groups/participants free of the outcome at the start of the study (or at the moment of exposure)? **Q7**: Were the outcomes measured in a valid and reliable way? **Q8**: Was the follow up time reported and sufficient to be long enough for outcomes to occur? **Q9**: Was follow up complete, and if not, were the reasons to loss to follow up described and explored? **Q10**: Were strategies to address incomplete follow up utilized? **Q11**: Was appropriate statistical analysis used?
